# The Use of Natural Sorbents in Cow Feed to Reduce Gaseous Air Pollutants and Faecal Biogenic Compounds

**DOI:** 10.3390/ani15050643

**Published:** 2025-02-22

**Authors:** Bożena Nowakowicz-Dębek, Łukasz Wlazło, Jolanta Król, Katarzyna Karpińska, Mateusz Ossowski, Hanna Bis-Wencel, Wojciech Ospałek

**Affiliations:** 1 Department of Animal Hygiene and Environmental Hazards, Faculty of Animal Sciences and Bioeconomy, University of Life Science in Lublin, Akademicka 13, 20-950 Lublin, Poland; bozena.nowakowicz@up.lublin.pl (B.N.-D.); lukasz.wlazlo@up.lublin.pl (Ł.W.); hanna.biswencel@up.lublin.pl (H.B.-W.); wojciech.ospalek@up.lublin.pl (W.O.); 2 Department of Quality Assessment and Processing of Animal Products, Faculty of Animal Sciences and Bioeconomy, University of Life Science in Lublin, Akademicka 13, 20-950 Lublin, Poland; jolanta.krol@up.lublin.pl

**Keywords:** dairy cows, gaseous pollutants, greenhouse gases, biogenic substances, natural sorbents, environment

## Abstract

The implementation of new technologies and best practices on factory farms is crucial for reducing the environmental pollution burden. This study aimed to evaluate the use of natural sorbents in the cows’ diet to reduce gaseous pollutants released on the farm and the content of faecal biogenic compounds. To this end, a mixture of natural sorbents (65% beechwood biochar, 25% aluminosilicate, and 10% glycerine) was added to cows’ feed. Pollutants released from all groups of cows were continually monitored on the farm during the experiment. Blood samples were also collected from the cows for haematological and biochemical analysis to determine the sorbents’ impact on their health. The average level of gaseous pollutants in the air on the farm were highest in the control group and lowest in the experimental groups. The levels of NH_3_, CH_4_, and H_2_S were statistically significant at *p* < 0.05. The results demonstrated that the added sorbents effectively reduced gaseous pollutants without adversely affecting the health of cows. Natural additives in the cows’ diet, including sorbents, bind harmful substances such as NH_3_ and CH_4_, which are common gaseous by-products of digestion. This leads to improvements in animal welfare and the natural environment.

## Simple Summary

This study investigated the impact of incorporating natural sorbents into cow feed for the reduction of gas emissions (ammonia, methane, and hydrogen sulfide) and the content of faecal biogenic compounds. Cows were divided into three dietary groups: a control group receiving a standard diet (C), a group supplemented with 0.25% of the sorbent mixture (E1), and a group supplemented with 0.5% of the sorbent mixture (E2). The highest concentrations of the analysed gases were detected in the control group, whereas the lowest concentrations were observed in the E2 group. Minimal variations in blood parameters were recorded among the groups; however, triglyceride and urea levels were highest in the control group. Microbial analysis of the feed and faeces revealed no significant correlations with the dietary treatments. The results demonstrated that sorbents effectively reduce pollutant emissions without adversely affecting animal health. Thus, the inclusion of sorbents in cow feed has the potential to enhance animal welfare while mitigating environmental pollution.

## 1. Introduction

European countries are required to report emissions of greenhouse gases (GHGs) and other gaseous pollutants, an obligation arising from international commitments to climate protection [1]. In accordance with these provisions, data on GHG emissions across various sectors of the economy are systematically collected and analysed. One of the main sources of pollutant emissions is the agricultural sector, particularly cattle, pig, and poultry farms, which account for approximately 60% of ammonia (NH_3_) emissions and 43% of methane (CH_4_) emissions from animal production in the EU [2,3,4]. Cattle farms are responsible for nearly 40% of the inventory of NH_3_ emissions [5,6]. The release of NH_3_ from farms is caused by an excessive nitrogen supply in animals feed, leading to excretion into the environment through faeces and urine. Both NH_3_ and CH_4_ are generated in the digestive tract (DT) of animals during bacterial fermentation, especially in ruminants [7]. Both of these pollutants pose significant environmental challenges, contributing to climate change. To mitigate agricultural pollution and other anthropogenic sources, the Directive (EU) 2016/2284 of the European Parliament and of the Council from 14 December 2016 on the reduction of national emissions of certain atmospheric pollutants’ (NEC Directive) aims to regulate emissions of harmful substances, including NH_3_ and CH_4_, [1]. The NEC Directive mandates that member states develop and implement national programmes to reduce emissions to specified limits. Poland’s emission reduction obligations are divided into two periods: from 2020 to 2029 and from 2030 onward. For NH_3_, these reductions are set at 1% annually from 2020 to 2029 and 17% annually from 2030, based on 2005 reference levels. According to the NEC Directive, the key tools for effective NH_3_ emission reductions from agriculture include the measures and strategies outlined in the National Air Pollution Control Programme, as well as voluntary principles in the National Code of Good Agricultural Practice for reducing NH_3_ emissions. In Poland, the national programme for reducing pollutants in the country defines strategies and actions aimed at minimising emissions of NH_3_, CH_4_, and other pollutants. These strategies encompass investments in technologies, modifications to agricultural practices, and slurry management, among other measures. Such interventions not only reduce agriculture’s impact on climate change but also protect the air, soil, and groundwater quality, and both human and animal health [8]. Implementing appropriate agricultural practices, technologies, and policies is crucial to achieving environmental protection and sustainable agriculture goals [9].

Due to their unique ion exchange properties and the ability to irreversibly bind and release water, as well as absorb substances with specific cross-sectional diameters, there has been growing interest in using natural and synthetic sorbents in cow feeding. These sorbents are explored for their potential to reduce mycotoxins, and to lower NH_3_ levels in the rumen. They are currently used mainly to improve performance characteristics and prevent some metabolic diseases in dairy cattle [10]. Another reason for incorporating zeolites into feed is their ability to reduce NH_3_ emissions in manure [11]. By utilising the adsorption capacity of mineral sorbents, undesirable gases and endotoxins can be removed from the DT, thereby improving feed digestibility and preventing diarrhoea. The inhibitory effects on harmful bacteria in the DT are attributed to changes in hydrogen ion concentrations caused by the ion exchange action of bentonite [12]. The improved protein digestibility is due to the absorption of NH_3_ molecules by bentonite and their gradual release along the DT, enhancing nitrogen utilisation [13]. Studies have demonstrated that the addition of bentonite or montmorillonite at 20 g/kg of dry matter (DM) concentrate in lactating cows’ diets improves nutrient digestibility and milk composition while reducing aflatoxin M1 (AFM1) aflatoxin content in milk. This translates to the improved health properties of milk for consumers [14]. The literature on natural sorbents in dairy cows’ diet predominantly focuses on zeolites and bentonite, with limited studies on biochar. Biochar, widely utilised as a feed additive in poultry production, has attracted interest in intensive dairy production due to its sorption properties [15]. Biochar is produced during the pyrolysis of various types of organic material (biomass) at high temperatures ranging from 350 to 1000 °C, in an environment with zero or low oxygen content [16]. The end products of the process include gaseous compounds, such as water vapor, carbon dioxide (CO_2_), carbon monoxide (CO), hydrogen (H_2_), CH_4_, ethane (C_2_H_6_), and a solid fraction containing large amounts of carbon. The carbon residues from this process are referred to as “biochar”. Biochar is most commonly produced from the by-products of agricultural, industrial, or waste management processes—such as hay, rice husks, wood chips, grass and cereal residues, animal manure, or sewage sludge. These materials are typically classified as waste that requires disposal, and their use for biochar production can therefore be considered a novel upcycling method [17]. Biochar has been shown to offer multiple benefits, including improved nutrient efficiency, contaminant absorption, and improved animal health following oral administration [18].

The aim of this study was to evaluate the addition of natural sorbents in cow feed on gaseous pollutants emissions (NH_3_, CH_4_, and H_2_S), macroelement concentrations, and faecal biogenic compounds.

## 2. Materials and Methods

The experiment was conducted on a dairy farm with the approval of the Local Ethical Committee (no. 72/2021) of the University of Life Sciences in Lublin, Poland. During the study, the farm, located in the southeastern region of the country, housed approximately 400 cows. A total of 150 Holstein-Friesian dairy cows were directly involved in the experiment and were divided into two experimental groups (E1 and E2) and a control group (C). The cows were assigned to experimental groups based on the similarity of characteristics, such as comparable health status, similar stages of lactation, number of lactations, and other related factors. Following calving, the cows remained in their respective technological groups for up to 150 days of lactation. The cows in the experimental groups (E1 and E2) were fed a diet supplemented with 0.25% and 0.5% of the sorbents mixture, respectively, while the control group (C) received a standard diet. The sorbent mixture comprised approximately 65% beechwood biochar, 25% aluminosilicate as an anticaking agent, and 10% glycerine as an antidusting agent. Prior to the farm experiment, laboratory analyses were conducted [19] (Table 1). This study employed a cross-over experimental design, divided into experimental periods in which each cow served as its own control. Cows within the groups were rotated through the experimental setup (control and experimental conditions). The control group (C) was used first in the experiment for a period of one month. Subsequently, the tested sorbents mixture was introduced into the cows’ diet (group E1). The experiment with this group was conducted for another month, followed by a two-week wash-out period to eliminate the experimental factor. Next, the proportion of the supplement in the cows’ diet was increased (Group E2), and a further one-month experimental period was carried out.

Milking was performed using a robotic milking system with an average of 2.8–2.9 visits/cow/day. The average duration of each visit, including washing, milking, and teat closure was approximately 7 min 30 s. The cows were kept on shallow bedding with access to drinking water and a feed table, where they were provided with a total mixed ration (TMR) formulated to support an average milk yield of 39–40 L/cow. The TMR, consisting of approximately 64 kg/cow/day, was supplied twice daily (morning and afternoon). The energy required to sustain milk production was supplemented with concentrated feed provided through the milking robot, which was tailored to the lactation stage and the individual yield of each cow. The average milk yield in the herd during the experiment ranged from 35 to 37 L/cow/day. In group E1, the annual (rolling) milk yield of the herd during the study period was 1722 kg/day. Notably, this group experienced a 62 kg decrease in total milk production compared to the corresponding period in the previous year. In contrast, group E2 achieved an annual (rolling) milk yield of 1810 kg/day during the study period, representing an increase in 91 kg relative to the corresponding period in the previous year. The fat content ranged from 3.38% (group C) to 3.96% (E2), and the protein content from 3.44% in control group to 3.64% in group E2 (Table 2).

During the experimental period, the cows were subjected to milk performance evaluation by the Polish Federation of Cattle Breeders and Dairy Farmers. The AR4 evaluation method, specifically designed for milking robots, was employed for the herd, with at least 11 test days/year, at four-week intervals. This enabled the collection of information on the daily yield of milk and its composition based on data from the robot’s computer system and a milk sample automatically collected for each cow from one milking session per day.

### 2.1. Analysis of the Concentrations of Gases from Cattle Farm Buildings

**Figure 1:**
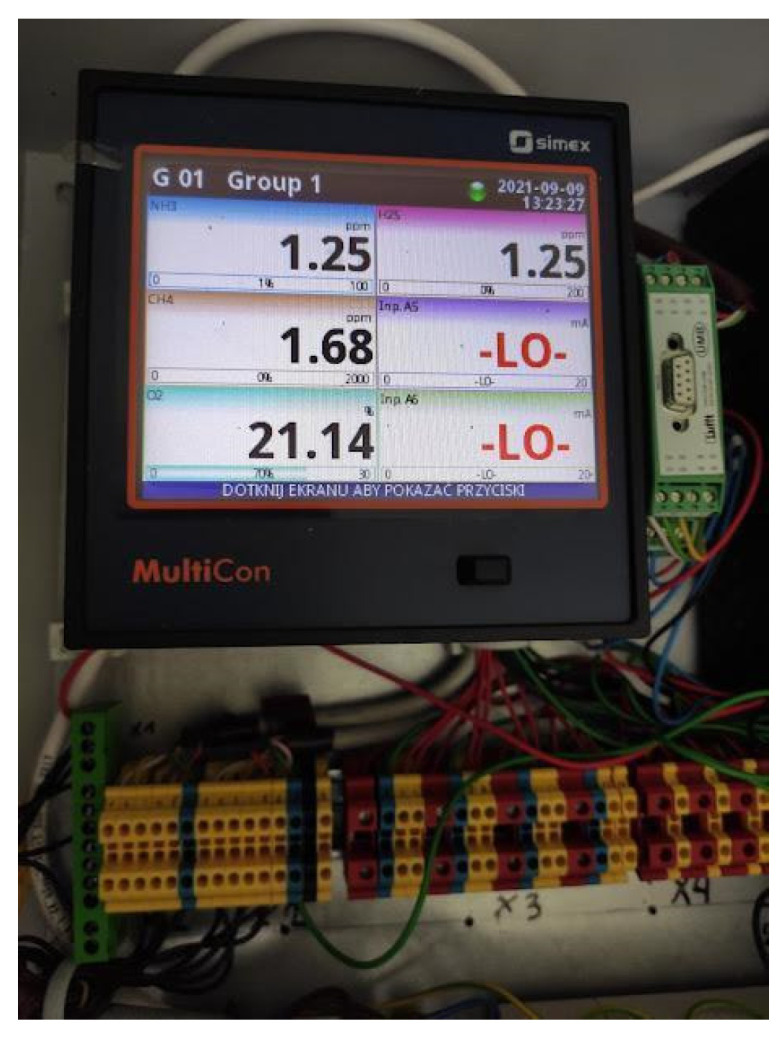
Fresenius GA 220 Multigas Analyser.

The concentrations of the gases in the cowshed in which the animals were present during the experiment were measured using the Fresenius GA 220 Multigas Analyser (Fresenius Umwelttechnik GmbH., Herten, Germany), (Figure 1). The analyser consisted of non-dispersive infrared (NDIR) gas sensors equipped with electrochemical sensors for the determination of NH_3_, CH_4_, H_2_S, and O_2_ concentrations, along with a reference system. The characteristics of the NDIR sensors are presented in Table 3 [20].

**Figure 2:**
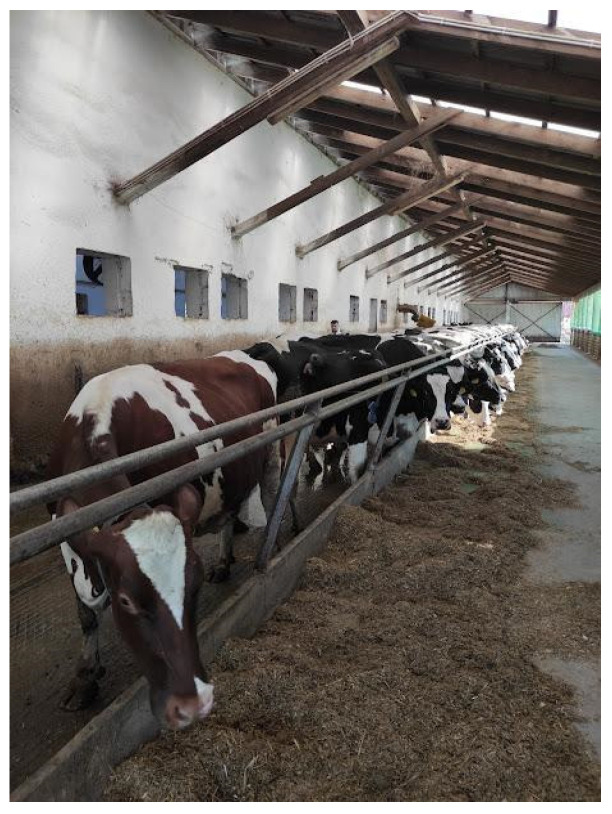
Animal housing conditions/feed table.

Air for analysis was sampled using a pump with a polytetrafluoroethylene tube (6 mm diameter). Six measuring points were selected on the farm:

1. milking robot;
2. corridor outside the milking robot exit;
3. cows’ resting area;
4. passage between the resting area and the feed table;
5. in front of the feed table;
6. feed table (Figure 2).

More than 180 measurement cycles of the gaseous pollutants were recorded per day at all measuring points. At the end of the experiment, the percentage reduction in released pollutants (NH_3_, CH_4_, and H_2_S) was calculated according to the following Formula (1), [19]:

Re = 100% − [(CG × 100%/CC)],(1)

where: Re—NH_3_, CH_4_ and H_2_S reduction (%); CG—the average amount of NH_3_, CH_4_, and H_2_S released in the experimental group; and CC—the average amount of NH_3_, CH_4_ and H_2_S released in the control group.

Microclimatic parameters were monitored on the farm using temperature and relative humidity sensors within an integrated system with the Fresenius GA 220 analyser.

### 2.2. Sampling of Biological Material for Analysis

Samples of the feed (n = 6) were collected immediately after feeding the cows, directly from the feeding table. Samples of the cows’ faeces were collected in sterile containers from 6 cows from each group immediately after the excretion. All samples were immediately chilled to 4–8 °C and transported to the laboratory. Subsequently, a Class 2 safety cabinet (Thermo Scientific, Waltham, MA, USA) was used to collect 20 g samples of each material. These samples were then homogenised using a CAT Unidrive X1000 homogeniser (Deerfield, IL, USA) and subjected to microbiological analysis. To monitor the health of the animals during the experiment, six cows from each group, (as approved by the Local Ethical Committee No. 72/2021), matched by body weight and lactation number, were selected for blood analysis. The selected animals exhibited no clinical pathological changes in the mammary gland or hoof horn and were in good general health. Analysis of the somatic cell count obtained from the milking robot data indicated no subclinical inflammation of the udder. Blood was drawn in the morning from the jugular vein, following local anaesthesia, into Monovette clot activator tubes (Sarstedt Inc., Nümbrecht, Germany). The blood was transported to the laboratory in thermal bags at approximately 4 °C. For biochemical analysis, the blood was centrifuged (3000 rpm for 10 min) in the laboratory to obtain plasma.

### 2.3. Analysis of Macroelements and Biogenic Substances in Cow Faeces

Faecal samples from animals in all groups were analysed for DM content (%), sodium (Na), total nitrogen (N), phosphorus (P and P_2_O_5_), potassium (K and K_2_O), calcium (Ca), and magnesium (Mg). The samples were mineralised using sulfuric acid and hydrogen peroxide. The K content was determined by flame photometry using a SHERWOOD flame photometer (Sherwood Scientific Ltd., Cambridge, UK). The P content was quantified by vanadomolybdate spectrophotometry using a Genesis 6 spectrophotometer (Thermo Fisher Scientific Inc., Waltham, MA, USA). The N concentration was determined by titration using an N KjelFlex K-360 system. Microelements and biogenic substances were analysed following procedures in an accredited laboratory.

### 2.4. Microbiological Analysis

From each of the homogenised feed and faecal samples, 20 g of material was weighed and placed in sterile bottles containing Ringer’s solution. The analytical procedure is described in detail by Wlazło et al. [21]. The material was analysed using media from BTL Ltd., Łódź, Poland, and BioMerieux, Ltd., Warsaw, Poland, for the following parameters:

1. Total number of aerobic mesophilic bacteria;
2. Total number of fungi;
3. Total number of coliform bacteria;
4. Total number of *Escherichia coli*;
5. Total number of *Clostridium perfringens*;
6. The total number of lactic acid bacteria (LAB) of the genus *Lactobacillus*;
7. The presence of *Salmonella*.

Each feed and faecal sample was plated in triplicate and incubated as described by Wlazlo et al. [21]. The resulting colonies were counted using an automatic colony counter Scan 300 (Interscience, Saint Nom la Brétèche, France) and converted to colony forming units per gram of feed or faeces (cfu/g) in accordance with ISO 4832 [22] and EN ISO 7218 [23].

### 2.5. Analysis of Animal Blood

Basic haematological analyses, including red blood cell count (RBC), haematocrit (HCT), haemoglobin content (HGB), white blood cell count (WBC), and leukogram, were performed on whole blood from the cows using a 5-diff BC-5000 Vet analyser (Mindray Animal Medical Technology Co., Ltd., Shenzhen, China), which operates on the principle of flow cytometry. The blood plasma was analysed for biochemical parameters, including alanine aminotransferase (ALT), aspartate aminotransferase (AST), total bilirubin (BIT), calcium (CAV), creatine kinase—non-vascular (CKNV), creatinine (CREAV), glucose (GLU), magnesium (MGV), phosphorus (PHO), total protein (PROV), triglycerides (TG), and urea (UREAV). These analyses were conducted using the ROCHE Cobas c311 automatic biochemical analyser with an ISE module (Roche Diagnostics Ltd., Warszawa, Poland). Electrolytes (Na, K, and Cl) were determined via direct potentiometry, while the remaining biochemical parameters were measured using photometric methods.

### 2.6. Statistical Analysis

The results were presented as the arithmetic mean (M) from each group (C, E1, and E2), standard error of the mean (SEM), and *p*-value. The statistical analysis was performed using Statistica software version 13.1 (StatSoft Inc., Tulsa, OK, USA). The normality of the distribution was tested by Levene’s test. If the distribution was normal, analysis of variance (ANOVA) and post-hoc tests were performed. Means in columns between rows marked with different letters (a, b…) were significantly different at *p* < 0.05.

## 3. Results

The results for the concentration of the gaseous pollutants at all sampling points on the farm are presented in Table 3, Table 4, Table 5 and Table 6. The NH_3_ concentration for the control group (C) and group E2 was highest at point 1 (milking robot), while for E1 it was highest at point 3 (cows’ resting area). The values were lowest at point 6 (feed table) in all groups. None of the values exceeded the acceptable limit of 15.4 mg/m^3^, which is particularly important in protecting the environment against reactive oxygen species. All values were statistically significant at *p* = 0.000 (Table 4).

Methane is produced in the rumen of cows through the activity of methanogenic bacteria. On the farm where the study was conducted, CH_4_ concentration was highest in the control group (C) at all sampling points. The highest concentration across all groups was observed at point 1 (milking robot), while the lowest was observed for groups C and E2 at point 6 (feed table) and for E1 at point 5 (in front of the feed table). All values were statistically significant at *p* = 0.000 (Table 5).

The next identified pollutant was H_2_S, which is generated in buildings by the decomposition of protein-rich fermented substances. The H_2_S concentration in the air was highest in the control group at all sampling points except for point 5 (in front of the feed table), where the highest value was recorded for E1. The highest overall concentration for all groups was observed at point 6 (feed table). All concentrations were below the acceptable limit of 7.5 mg/m^3^. Statistically significant differences were found only at points 5 (in front of the feed table) and 6 (feed table) at *p* = 0.000 (Table 6).

The average levels of gaseous pollutants in the air of the farm were highest in the control group and lowest in group E2. The NH_3_, CH_4_, and H_2_S concentrations were all statistically significant at *p* = 0.000 (Table 7).

Diagnosis of the health condition of the cows was based on the peripheral blood cell parameters. The WBC count was comparable across all groups. Statistically significant differences were observed for BAS at *p* = 0.032%. All parameters were found to be within the reference ranges (Table 8).

The red blood cell parameters of the cows included in the experiment are presented in Table 9. Except for MCHC, all parameters exhibited similar trends across groups and were within reference ranges. The average MCHC was statistically significantly higher in group E2, at *p* = 0.001. The MCHC value is expressed as a percentage of the total HGB content. This parameter provides insight into the current state of blood cells and indicates the extent to which erythrocytes are saturated with HGB. It can also increase under conditions of dehydration; however, this problem is unlikely to occur when animals have constant access to water.

The results for the activity of the liver enzymes and other biochemical parameters of the blood serum of the cows are presented in Table 9. The activity of aminotransferases was not significantly higher in the control group and exceeded the reference values. The high CKNV level indicated the body’s increased energy demand. The activity of this enzyme also slightly exceeded the reference values reported by Winnicka et al. [24]. Triglycerides produced in the liver are a form of fat reserves in the body. The highest TG level was obtained in the control group (17.62 mg/dL), and it was statistically significantly higher than in the experimental groups, at *p* < 0.000. Urea is a product of protein breakdown, and its concentration in plasma provides information about the functioning of the rumen and liver, as well as the intake of this substance in the diet. This parameter was similar in the control group C and group E1, while in group E2, it was statistically significantly higher at *p* < 0.05. A similar pattern was noted for BIT, GLU, and Na, and these values were statistically significant at *p* < 0.05 (Table 10).

The DM content of the cow faeces ranged from 14.50 (E1) to 14.75% (C) and was not statistically significant at *p* < 0.05. The faeces from the control group had a significantly higher content of P (*p* < 0.05) and Mg, at 0.11% and 0.13%, respectively (Table 11).

Yeasts are involved in the enzymatic processes taking place in feed and are responsible for its oxidation. Therefore, the microbiological control of feed is crucial to both sanitation and nutrition. Table 12 presents the numbers of microorganisms in the cows’ feed during the study period. The number of aerobic mesophilic bacteria was highest in group E2 (2.3 × 10^7^ cfu/g) and lowest in the control group (2.0 × 10^4^ cfu/g). The concentration of fungi in the feed was similar in all groups. However, the number of *Clostridium perfringens* in the feed was slightly higher in group E1 (2.7 × 10^4^ cfu/g). This group also had slightly lower numbers of LAB of the genus *Lactobacillus* (4.0 × 10^6^ cfu/g).

In the faeces of cows, the highest number of aerobic mesophilic bacteria was found in group E1 (1.6 × 10^7^ cfu/g). The faecal samples from this group also exhibited a slightly lower fungal count (8.5 × 10^5^ cfu/g) compared to the other groups. The faecal samples from the control group had the lowest number of *Clostridium perfringens* (1.4 × 10^4^ cfu/g), while the number of LAB of the genus *Lactobacillus* was lowest in the faeces of group E1 (7.6 × 10^4^ cfu/g). The concentrations of *E. coli* and coliform bacteria were similar across all groups. No bacteria of the genus *Salmonella* were identified in either the feed or the faecal samples (Table 13). The results of the microbiological analyses did not indicate any dependence on the feed additive. They should be considered as a statistical distribution of values in the samples, except for the total number of aerobic mesophilic bacteria, where a statistically significant difference (*p* < 0.05) was observed.

## 4. Discussion

The effects of natural biogenic substances such as N and P on the environment represent an increasing challenge for animal production, in terms of both the technologies used and the cost intensity. In response to the contribution of agricultural pollutants to global pollution levels, animal production has become subject to several regulations aimed at protecting the natural environment. At the EU level, this primarily refers to the directive concerning the protection of water resources from pollution caused by nitrates of agricultural origin. The main recommendations for cattle farmers include methods of precision feeding for farm animals [25]. 

These methods involve reducing the level of crude protein (CP) in feed by 15–20%, which raises concerns among farmers about a potential decrease in production. Kasprowicz-Potocka and Frankiewicz [26] demonstrated that this measure did not adversely affect the production of fattening pigs and reduced the N emission to the environment, but it required additional feed supplementation with essential amino acids (AA). The impact of modern milk production systems on the environment and efforts to reduce the CP level in the diet of dairy cattle are currently the subjects of research at many research centres. According to Sinclair et al. [27], there is a strong positive correlation between CP concentration and DM intake. This is particularly evident in the CP range of 140 and 220 g/kg DM. However, these effects are modest, and the reduction in DM intake can be partially compensated by improving the digestibility and AA profile of undegradable protein (UDP) as a component of the diet or by increasing fermentation in the rumen. Low N retention in dairy products (approximately 25%) and high losses of the remaining N in the urine and faeces necessitate the search for alternative, more cost-effective plant-based protein sources or non-protein N sources [28]. According to Sinclair et al. [27], there is a need for feeding programmes that will enable N retention in the udder, improving digestion and the absorption of biogenic substances in the lower intestine. The present study is in line with this strategy and demonstrates that the inclusion of biochar in the test diets reduced the level of P excreted in the faeces of animals from the experimental group without adversely affecting their health. 

The effect of biochar in the animals’ diet has also been investigated by Prasai et al. [29] According to the authors, a 4% addition of biochar, bentonite, and zeolite to chickens’ diets can selectively control pathogenic intestinal microorganisms. The research showed that feeding chickens a diet enriched with these additives was associated with a reduction in the number of Gram negative bacteria, i.e., *Helicobacter* and *Campylobacter*, which are among the major pathogens in poultry. At the same time, they were not shown to reduce the richness and diversity of the intestinal microbiota compared to the control group, which may indicate that these compounds could potentially be used as an alternative to antibiotics in the poultry industry. The present study also showed no significant effect of the tested biochar levels in the cows’ diet on the composition and number of microorganisms in the feed. This is supported by a study by Goiri et al. [30] investigating the effect of biochar in chickens’ diet. The authors reported a decrease in the number of *Clostridium* sp. and *Lactobacillus* sp. in the intestines of the animals whose diet was supplemented with biochar, which also was not observed in the present study. Contrasting results were obtained by Chu et al. [31], who fed pigs a diet with bamboo biochar. The intestinal microbiota of the animals receiving this additive had more anaerobic bacteria and LAB, and fewer coliform and *Salmonella* bacteria. The present study showed no change in the number of *E. coli* in the animals’ faeces whose diets were supplemented with beechwood biochar and aluminosilicates. Other authors [32], however, have demonstrated that a 1% addition of rice husk biochar to chickens’ diet reduced the presence of *E. coli* in their faeces.

Biochar, due to its high porosity, contributes to the creation of a specific environment for the growth of methanogenic microorganisms and positively influences microbiological fermentation processes, which translates directly into a reduction in CH_4_ emissions by ruminants, which are a major source of gaseous pollutants [33]. This was confirmed in the present study, in which the additive tested was shown to have properties reducing the amount of gaseous pollutants released. Natural additives in the diet of cows, including sorbents, are aimed at reducing pollution generated by these animals, which translates into improvements in animal welfare and the natural environment [34]. Sorbents such as biochar, bentonite clay, or zeolite can be added to cow feed to bind harmful substances such as NH_3_ and CH_4_, which are common gaseous by-products of digestion [33,35]. The present study confirmed the value of using natural feed additives, which significantly reduces the environmental burden. Khachlouf et al. [10] showed that the addition of zeolite to cow feed not only reduced the level of NH_3_ but also increased milk yield. Similar results were obtained in the present study. Similar studies have been conducted for other animal species, such as pigs and chicken broilers [36,37,38,39]. Natural sorbents are often used in research as feed additives due to their effectiveness and commercial availability. They help to reduce emissions by absorbing and neutralizing harmful substances in animal faeces.

Apart from environmental benefits, a reduction in the emissions of harmful gaseous pollutants also has public health and economic benefits. There is a need to reduce the emissions of NH_3_, which can lead to the formation of PM2.5 and PM10 particles as well as volatile organic compounds (VOCs), which are precursors of smog. These measures help to improve air quality and reduce negative effects on the climate [40,41]. According to Martínez-Marín et al. [42], CH_4_ emissions, and in particular their variation during lactation, can be estimated using the infrared spectroscopy of milk. The milk parameters predicted by the authors were 3.79% fat and 3.68% protein, and the daily CH_4_ production was estimated at 358 g/d. These relationships were not explored in the present study, which was based on actual measurements. The fat content in milk ranged from 3.38% in the control group to 3.96% in group E2, and in the latter group, it was slightly higher than the values estimated by Martínez-Marín et al. [42]. In the case of protein content, the values observed in the present study were slightly lower. However, the authors stress that the modelling of lactation does not explain the mechanism of CH_4_ release, which remains at a constant level. According to Tongwane et al. [43], the problem of CH_4_ emissions on cattle farms also affects South Africa, with the volume of emissions depending on multiple factors, including the category of cattle, their feed, its digestibility, and the housing system. Hence there is a need to develop emission factors for waste management on the farm for different categories of cattle. 

Appuhamy et al. [44] suggested that it is useful to create models associated with the production of biodegradable and non-biodegradable volatile substances (VS) from manure. According to the Intergovernmental Panel on Climate Change (IPCC), CH_4_ released from manure piles is determined based on VS. Therefore, mathematical models for VS could improve the estimation of CH_4_ emissions from faeces. Models recommended by the IPCC require the precise determination of feed digestibility, which is often difficult to do at the farm level. Thus, it seems that the focus must be placed on reducing the impact of the farm on the external environment using measures that involve maximizing the use of all pollutants within the farm itself. In this way, GHG emissions are already eliminated at the farm level [45].

Investment in technologies and practices aimed at reducing the emissions of harmful chemical substances from cow faeces, such as the use of sorbents as additives, is crucial for protecting both the environment and public health. These measures contribute to the creation of more sustainable animal production systems following the European Parliament resolution from 21 October 2021 on an EU strategy to reduce CH_4_ emissions [45]. The spread of extensive production is not a solution to the problem, as it increases CH_4_ emissions per unit of production. According to Liu et al. [46], strict regulations require an emissions inventory based on reliable emission factors. Thus, there is a need for a suitable strategy to reduce GHG emissions that will decrease the environmental footprint while maintaining production quality.

## 5. Conclusions

The proposed additives in the cows’ diet had positive effects, reducing air pollutants in both experimental groups. It is particularly worth noting that the level of CH_4_ decreased by nearly 30% compared to the control group. Moreover, the use of natural sorbents in the diet did not adversely affect the animals’ health, as confirmed by blood tests. The results suggest that the sorbents achieved their intended purpose, i.e., to reduce gaseous pollutants in the form of NH_3_, CH_4_, and H_2_S, which translates into improved animal welfare and protection of the natural environment.

## Figures and Tables

**Table 1:** Feed ration during the study period (kg of feedstuffs).

Ingredient	DM (%)	kg of Product	kg DM
Maize silage	36	24.0	8.6
Alfalfa silage	47	6.0	2.8
Gorzowska grass mixture (50% Italian ryegrass, 30% crimson clover, 20% winter vetch)	37	4.0	1.5
Brewer’s spent grain	22	10.0	2.2
Sugar beet pulp	24	7.0	1.7
Water	0	4.0	0.0
Wheat	86	2.8	2.4
Rapeseed meal	88	2.5	2.2
Mineral and vitamin premix	95	0.50	0.47
DM (%)	36–38		
DM intake kg/animal/day	21.2		

DM—dry matter.

**Table 2:** Production parameters of the cows in each group during the study.

Parameter	Group		
	C	E1	E2
Cows (*n*)	50	51	49
Day of lactation (average)	161	179	185
Milk (kg/day)	35.6	33.8	36.9
Fat (%)	3.38	3.53	3.96
Protein (%)	3.44	3.51	3.64
Casein (%)	2.70	2.71	2.82

Groups: C—control group; E1—cows receiving a diet with 0.25% sorbent mixture (65% beechwood biochar, 25% aluminosilicate, and 10% glycerine); E2—cows receiving a diet with 0.5% sorbent mixture (65% beechwood biochar, 25% aluminosilicate, and 10% glycerine).

**Table 3:** Non-dispersive infrared (NDIR) gas sensors characteristics.

Parameter	Measurement Range	Measurement Accuracy	Detection Limits
Methane (CH_4_)	0–5000 ppm	2 ppm	3%
Ammonia (NH_3_)	0–1000 ppm	2 ppm	3%
Hydrogen sulfide (H_2_S)	0–10,000 ppm	2 ppm	5%
Oxygen (O_2_)	0–30 vol%	0.1 vol%	5%

**Table 4:** Concentration of NH_3_ at each sampling point on the farm (mg/m^3^).

Sampling Site	C	E1	E2	M	SEM	*p*-Value
Point 1	2.66 ^b^	2.35 ^a,c^	2.68 ^c^	2.56	0.017	0.000
Point 2	2.52 ^a^	2.30 ^d^	1.59 ^b,c^	2.18	0.016	0.000
Point 3	2.24 ^a^	2.52 ^a,c^	1.41 ^b^	2.01	0.016	0.000
Point 4	2.21 ^a^	1.92 ^c^	1.28 ^b^	1.85	0.016	0.000
Point 5	2.06 ^a^	1.46 ^c^	1.19 ^b^	1.60	0.013	0.000
Point 6	1.97 ^a^	1.38 ^c^	1.08 ^b^	1.51	0.013	0.000

Sampling site: point 1—milking robot; point 2—corridor outside the milking robot exit; point 3—cows’ resting area; point 4—passage between resting area and feed table; point 5—in front of feed table; point 6—feed table. Groups: C—control group; E1—cows receiving a diet with the 0.25% sorbent mixture (65% beechwood biochar, 25% aluminosilicate, and 10% glycerine); E2—cows receiving a diet with the 0.5% sorbent mixture (65% beechwood biochar, 25% aluminosilicate, and 10% glycerine). M—mean; SEM—standard error of the mean; ^a,b,c,d^—values marked with different letters differ statistically significantly at *p* < 0.05.

**Table 5:** Concentration of CH_4_ at each sampling point on the farm (mg/m^3^).

Sampling Site	C	E1	E2	M	SEM	*p*-Value
Point 1	28.12 ^b^	21.83 ^a,c^	21.67 ^a,c^	24.02	0.200	0.000
Point 2	24.79 ^a^	18.02 ^c^	20.39 ^b^	21.12	0.195	0.000
Point 3	20.61 ^a^	19.99 ^a,c^	14.03 ^b^	18.57	0.207	0.000
Point 4	20.16 ^a^	15.37 ^c^	11.56 ^b^	16.05	0.200	0.000
Point 5	19.69 ^a^	11.01 ^b,c^	11.25 ^b^	14.20	0.196	0.000
Point 6	19.61 ^a^	13.67 ^c^	10.58 ^b^	14.96	0.211	0.000

Sampling site: point 1—milking robot; point 2—corridor outside the milking robot exit; point 3—cows’ resting area; point 4—passage between resting area and feed table; point 5—in front of feed table; point 6—feed table. Groups: C—control group; E1—cows receiving a diet with 0.25% sorbent mixture (65% beechwood biochar, 25% aluminosilicate, and 10% glycerine); E2—cows receiving a diet with 0.5% sorbent mixture (65% beechwood biochar, 25% aluminosilicate, and 10% glycerine). M—mean; SEM—standard error of the mean; ^a,b,c^—values marked with different letters differ statistically significantly at *p* < 0.05.

**Table 6:** Concentration of H_2_S at each sampling point on the farm (mg/m^3^).

Sampling Site	C	E1	E2	M	SEM	*p*-Value
Point 1	1.58	1.52	1.56	1.55	0.009	0.124
Point 2	1.57	1.52	1.54	1.54	0.007	0.086
Point 3	1.56	1.54	1.54	1.55	0.010	0.561
Point 4	1.55	1.54	1.53	1.55	0.795	0.251
Point 5	1.55 ^a^	1.57 ^b^	1.53 ^a,c^	1.55	0.003	0.000
Point 6	1.71 ^a^	1.63 ^c^	1.56 ^b,c^	1.64	0.012	0.000

Sampling site: point 1—milking robot; point 2—corridor outside the milking robot exit; point 3—cows’ resting area; point 4—passage between resting area and feed table; point 5—in front of feed table; point 6—feed table. Groups: C—control group; E1—cows receiving a diet with 0.25% sorbent mixture (65% beechwood biochar, 25% aluminosilicate, and 10% glycerine); E2—cows receiving a diet with 0.5% sorbent mixture (65% beechwood biochar, 25% aluminosilicate, and 10% glycerine). M—mean; SEM—standard error of the mean; ^a,b,c^—values marked with different letters differ statistically significantly at *p* < 0.05.

**Table 7:** Mean concentration of the pollutants in the air of the cowshed (mg/m^3^; O_2_%).

Gas	C	E1	E2	M	SEM	*p*-Value
NH_3_	2.28 ^a^	1.94 ^c^	1.60 ^b^	1.96	0.007	0.000
CH_4_	22.19 ^a^	16.20 ^c^	15.62 ^b^	18.18	0.084	0.000
H_2_S	1.59 ^a^	1.55 ^c^	1.54 ^b,c^	1.56	0.007	0.000
O_2_	21.01	21.01	21.01	21.01	21.010	0.273

Groups: C—control group; E1—cows receiving a diet with 0.25% sorbent mixture (65% beechwood biochar, 25% aluminosilicate, and 10% glycerine); E2—cows receiving a diet with 0.5% sorbent mixture (65% beechwood biochar, 25% aluminosilicate, and 10% glycerine). M—mean; SEM—standard error of the mean; ^a,b,c^—values marked with different letters differ statistically significantly at *p* < 0.05.

**Table 8:** White blood cell parameters in the blood of cows.

Parameter	Unit	C	E1	E2	M	SEM	*p*-Value
WBC	10^3^/µL	9.36	9.12	10.13	9.54	0.440	0.649
BAS	%	0.100 ^a^	0.000 ^b^	0.033	0.044	0.017	0.032
NEU	%	38.43	38.17	40.07	38.89	1.548	0.877
EOS	%	4.33	5.22	4.87	4.81	0.769	0.906
LYM	%	54.78	54.02	52.02	53.61	2.042	0.865
MON	%	2.35	2.60	3.017	2.66	0.272	0.628

WBC—white blood cells; BAS—basophils; NEU—neutrophils; EOS—eosinophils; LYM—lymphocytes; MON—monocytes. Groups: C—control group; E1—cows receiving a diet with 0.25% sorbent mixture (65% beechwood biochar, 25% aluminosilicate, and 10% glycerine); E2—cows receiving a diet with 0.5% sorbent mixture (65% beechwood biochar, 25% aluminosilicate, and 10% glycerine). M—mean; SEM—standard error of the mean; ^a,b^—values marked with different letters differ statistically significantly at *p* < 0.05.

**Table 9:** Red blood cell parameters in the blood of cows.

Parameter	Unit	C	E1	E2	M	SEM	*p*-Value
RBC	10^6^/µL	7.36	7.53	6.69	7.19	0.217	0.257
HGB	g/dL	11.55	11.57	11.08	11.40	0.202	0.569
MCV	µm^3^	45.58	44.82	45.65	45.35	1.013	0.940
MCH	pg	15.80	15.57	16.70	16.02	0.346	0.392
MCHC	g/dL	34.67 ^b^	34.70 ^b,c^	36.58 ^a^	35.33	0.285	0.001
RDW-CV	%	20.62	20.62	20.93	20.72	0.348	0.921
RDW-SD	µm^3^	32.97	32.93	33.52	33.14	0.638	0.925
HCT	%	33.37	33.37	30.30	32.34	0.681	0.099
PLT	10^3^/µL	440.33	404.50	471.00	438.61	39.606	0.810
MPV	µm^3^	6.23	6.32	6.18	6.24	0.154	0.945
PDW	%	15.02	14.97	14.83	14.94	0.062	0.482
PCT	%	0.25	0.25	0.28	0.26	0.023	0.772

RBC—red blood cells; HGB—haemoglobin; MCV—mean corpuscular volume; MCH—mean corpuscular haemoglobin; MCHC—mean corpuscular haemoglobin concentration; RDW-CV—red cell distribution width—coefficient of variation; RDW-SD—red cell distribution width—standard deviation; HCT—haematocrit; PLT—platelets; MPV—mean platelet volume; PDW—platelet distribution width; PCT—plateletcrit. Groups: C—control group; E1—cows receiving a diet with 0.25% sorbent mixture (65% beechwood biochar, 25% aluminosilicate, and 10% glycerine); E2—cows receiving a diet with 0.5% sorbent mixture (65% beechwood biochar, 25% aluminosilicate, and 10% glycerine). M—mean; SEM—standard error of the mean; ^a,b,c^—values marked with different letters differ statistically significantly at *p* < 0.05.

**Table 10:** Biochemical parameters in the blood of cows.

Parameter	Unit	C	E1	E2	M	SEM	*p*-Value
ALT	U/L	39.57	37.52	32.87	36.63	2.122	0.438
AST	U/L	146.43	144.55	125.15	138.71	7.761	0.492
BIT	mg/dL	0.15 ^b,c^	0.13 ^b^	0.24 ^a^	0.18	0.016	0.004
CAV	mg/dL	10.00	10.40	10.83	10.41	0.199	0.242
CKNV	U/L	170.48	195.77	167.67	177.97	13.467	0.671
Cl	mmol/L	98.92	99.78	96.80	98.50	0.600	0.109
CREAV	mg/dL	1.09	1.09	1.15	1.11	0.024	0.627
GLU	mg/dL	62.12	71.13	61.95	65.07	1.761	0.040
K	mmol/L	4.19	4.09	4.00	4.09	0.060	0.474
MGV	mg/dL	2.58	2.87	2.53	2.66	0.071	0.087
Na	mmol/L	132.88 ^c^	135.87 ^b^	138.07 ^a^	135.61	0.572	0.000
PHO	mg/dL	5.34	5.06	6.75	5.71	0.339	0.086
PROV	g/dL	7.17	6.97	6.76	6.97	0.091	0.179
TG	mg/dL	17.62 ^a^	7.70 ^b^	8.55 ^b^	11.29	1.353	0.000
UREAV	mg/dL	33.38 ^b,c^	32.25 ^b^	46.65 ^a^	37.43	1.743	0.000

ALT—alanine aminotransferase; AST—aspartate aminotransferase; BIT—total bilirubin; CAV—calcium; CKNV—creatine kinase—non-vascular; Cl—chloride; CREAV—creatinine; GLU—glucose; K—potassium; MGV—magnesium; Na—sodium; PHO—phosphorus; PROV—total protein; TG—triglycerides; UREAV—urea. Groups: C—control group; E1—cows receiving a diet with 0.25% sorbent mixture (65% beechwood biochar, 25% aluminosilicate, and 10% glycerine); E2—cows receiving a diet with 0.5% sorbent mixture (65% beechwood biochar, 25% aluminosilicate, and 10% glycerine). M—mean; SEM—standard error of the mean; ^a,b,c^—values marked with different letters differ statistically significantly at *p* < 0.05.

**Table 11:** Macroelements and biogenic substances in the faeces of animals as the % of dry matter (DM) of the sample.

Parameter	C	SD	E1	SD	E2	SD	SEM	*p*-Value
DM%	14.75	0.07	14.50	0.08	14.68	0.21	0.046	0.012
Na	0.02	0.01	0.02	0.01	0.02	0.00	0.001	0.144
N	0.40	0.01	0.36	0.02	0.38	0.04	0.007	0.131
P	0.13 ^a^	0.00	0.11 ^b^	0.01	0.11 ^b,c^	0.01	0.004	0.025
P_2_O_5_	0.28	0.02	0.25	0.03	0.26	0.03	0.007	0.138
K	0.10	0.01	0.09	0.01	0.10	0.01	0.003	0.205
K_2_O	0.11	0.00	0.10	0.01	0.10	0.00	0.002	0.086
Ca	0.25	0.00	0.26	0.02	0.20	0.05	0.011	0.164
Mg	0.13 ^a^	0.00	0.12 ^b,c^	0.00	0.13 ^a^	0.00	0.002	0.031

DM%—dry matter percentage; Na—sodium; N—nitrogen; P—phosphorus; P_2_O_5_—phosphorus pentoxide; K—potassium; K_2_O—potassium oxide; Ca—calcium; Mg—magnesium. Groups: C—control group; E1—cows receiving a diet with 0.25% sorbent mixture (65% beechwood biochar, 25% aluminosilicate, and 10% glycerine); E2—cows receiving a diet with 0.5% sorbent mixture (65% beechwood biochar, 25% aluminosilicate, and 10% glycerine). SD—standard deviation; SEM—standard error of the mean; ^a,b,c^—values marked with different letters differ statistically significantly at *p* < 0.05.

**Table 12:** Number of microorganisms in the cows’ feed during the study (cfu/g).

Parameter	C	E1	E2	SEM	*p*-Value
Total number of aerobic mesophilic bacteria	2.0 × 10^4 a^	8.6 × 10^6 b^	2.3 × 10^7 b^	3,311,568	0.025
Total number of yeast-like fungi	2.2 × 10^6^	2.2 × 10^6^	1.6 × 10^6^	94,407.1	0.146
Total number of coliform bacteria	1.9 × 10^4^	9.6 × 10^4^	8.5 × 10^4^	3202.3	0.243
Total number of *Escherichia coli*	5.8 × 10^4^	3.5 × 10^4^	3.4 × 10^3^	3,301,006	0.160
Total number of *Clostridium perfringens*	6.9 × 10^3^	2.7 × 10^4^	3.5 × 10^3^	6872.5	0.060
Total number of LAB of the genus *Lactobacillus*	3.0 × 10^7^	4.0 × 10^6^	1.2 × 10^7^	10,437.8	0.068
The presence of *Salmonella*	n.d.	n.d.	n.d.	–	–

Groups: C—control group; E1—cows receiving a diet with 0.25% sorbent mixture (65% beechwood biochar, 25% aluminosilicate, and 10% glycerine); E2—cows receiving a diet with 0.5% sorbent mixture (65% beechwood biochar, 25% aluminosilicate, and 10% glycerine). n.d.—not detected. SEM—standard error of the mean; ^a,b^—values marked with different letters differ statistically significantly at *p* < 0.05.

**Table 13:** Number of microorganisms in the faeces of cows during the study (cfu/g).

Parameter	C	E1	E2	SEM	*p*-Value
Total number of aerobic mesophilic bacteria	4.9 × 10^6 a^	1.6 × 10^7 b^	8.9 × 10^6 b^	1,388,870	0.000
Total number of yeast-like fungi	2.6 × 10^6^	8.5 × 10^5^	2.0 × 10^6^	219,918.7	0.559
Total number of coliform bacteria	1.8 × 10^5^	1.2 × 10^5^	8.9 × 10^5^	60,227.7	0.195
Total number of *Escherichia coli*	7.7 × 10^5^	1.2 × 10^5^	8.6 × 10^5^	718,312.9	0.463
Total number of *Clostridium perfringens*	1.4 × 10^4^	1.2 × 10^5^	4.8 × 10^5^	99,663.4	0.220
Total number of LAB of the genus *Lactobacillus*	9.4 × 10^5^	7.6 × 10^4^	5.5 × 10^6^	105,588.6	0.142
The presence of *Salmonella*	n.d.	n.d.	n.d.	–	–

Groups: C—control group; E1—cows receiving a diet with 0.25% sorbent mixture (65% beechwood biochar, 25% aluminosilicate, and 10% glycerine); E2—cows receiving a diet with 0.5% sorbent mixture (65% beechwood biochar, 25% aluminosilicate, and 10% glycerine). n.d.—not detected. SEM—standard error of the mean; ^a, b^—values marked with different letters differ statistically significantly at *p* < 0.05.

## Data Availability

All relevant data are within the paper.
